# A De Novo *USP24* Variant as a Candidate Driver in a Neurodevelopmental Disorder: Insights from Trio-Based Whole-Exome Sequencing

**DOI:** 10.3390/ijms27094086

**Published:** 2026-05-02

**Authors:** Mirella Vinci, Antonino Musumeci, Simone Treccarichi, Miriam Virgillito, Siria Calì, Angelo Gloria, Concetta Federico, Salvatore Saccone, Maurizio Elia, Francesco Calì

**Affiliations:** 1 Oasi Research Institute-IRCCS, 94018 Troina, Italy; mvinci@oasi.en.it (M.V.); amusumeci@oasi.en.it (A.M.); agloria@oasi.en.it (A.G.); melia@oasi.en.it (M.E.); cali@oasi.en.it (F.C.); 2 Department of Biological, Geological and Environmental Sciences, University of Catania, 95124 Catania, Italy; virgillito.miriam@studium.unict.it (M.V.); siria.cali@studium.unict.it (S.C.); concetta.federico@unict.it (C.F.); salvatore.saccone@unict.it (S.S.); 3 Department Medicine and Surgery, Kore University of Enna, 94100 Enna, Italy

**Keywords:** next generation sequencing, molecular diagnosis, neurodevelopmental disorders, neurexin, ubiquitin carboxyl-terminal hydrolase, Low-density lipoprotein receptor-related protein

## Abstract

Neurodevelopmental disorders (NDDs), including autism spectrum disorder (ASD), are increasingly recognized as conditions with a complex, multisystemic origin. ASD frequently co-occurs with other neurological conditions, such as epilepsy. We report a female patient, born to unrelated healthy parents, presenting with a complex clinical phenotype characterized by ASD level 1 with fluent speech, borderline intellectual functioning (BIF), coordination disorder, and epilepsy. Trio-based whole-exome sequencing (WES) revealed a de novo variant in the *USP24* gene (c.3155G>T; p.Ser1052Ile), classified as likely pathogenic according to ACMG criteria (PS2, PM2, PP2, BP4). *USP24* has previously been associated with Parkinson’s disease and has recently emerged as a candidate risk gene for ASD. In addition, WES detected two variants of uncertain significance (VUS), both inherited from the clinically unaffected father: c.388G>C (p.Gly130Arg) in *NRXN2* and c.6395C>A (p.Ser2132Tyr) in *LRP2*. Although neither gene shows a fully penetrant causal relationship with the observed phenotype, both have been implicated in neurodevelopmental disorders. Array-CGH analysis did not reveal pathogenic copy number variants; however, the presence of additional genetic contributors not detectable by WES cannot be excluded. Overall, the de novo USP24 variant likely represents the primary genetic driver of the phenotype, while the potential contribution of the inherited *NRXN2* and *LRP2* variants remains plausible. This case underscores the complexity of the genetic architecture underlying NDDs and supports a model involving cumulative effects of multiple variants rather than a strictly multigenic interaction.

## 1. Introduction

Neurodevelopmental disorders (NDDs) comprise a heterogeneous group of conditions characterized by impairments in cognitive, behavioral, and motor functions arising during early brain development [1]. Among these, autism spectrum disorder (ASD) represents one of the most prevalent and clinically complex entities. According to the Diagnostic and Statistical Manual of Mental Disorders, Fifth Edition (DSM-5), ASD is defined by persistent deficits in social communication and social interaction, together with restricted, repetitive patterns of behavior, interests, or activities, with symptoms manifesting in early developmental stages [2,3]. Increasing evidence indicates that ASD and other NDDs have a multisystemic and multifactorial origin, resulting from the interplay of genetic and environmental factors. In addition to core behavioral features, individuals with ASD frequently present with comorbid conditions, including epilepsy, intellectual disability, and motor coordination disorders [4,5]. Notably, epilepsy is one of the most common neurological comorbidities in ASD, further supporting the involvement of widespread neurobiological mechanisms [6,7].

Advances in next-generation sequencing technologies, particularly whole-exome sequencing (WES), have significantly improved our understanding of the genetic architecture underlying NDDs [8,9]. These approaches have revealed that, in many cases, multiple rare variants affecting different genes may contribute to the phenotype, supporting a multifactorial or oligogenic model rather than a strictly monogenic cause [10,11]. Several studies have reported patients harboring more than one potentially relevant variant, suggesting additive or modifying effects across genes involved in neurodevelopmental pathways. The interpretation of genetic findings in NDDs is further complicated by phenomena such as variable expressivity and incomplete penetrance, whereby the same genetic variant may lead to different clinical manifestations or may be present in apparently unaffected individuals [12,13]. This is particularly relevant for variants inherited from healthy parents, which may still contribute to disease risk in combination with other genetic or environmental factors [14]. In parallel, de novo variants have emerged as major contributors to ASD and other NDDs. These variants, which arise spontaneously and are not inherited from either parent, are often enriched in genes involved in synaptic function, neuronal development, and chromatin regulation, and are more likely to have a strong functional impact.

Among the various molecular mechanisms implicated in NDDs, the ubiquitin–proteasome system (UPS) represents a pivotal regulatory pathway controlling protein homeostasis, synaptic function, and neuronal development [15,16]. This system relies on the coordinated activity of ubiquitin ligases and deubiquitinating enzymes (DUBs), which respectively add and remove ubiquitin moieties from target proteins, thereby modulating their stability, localization, and activity [17]. Increasing evidence has linked dysregulation of the UPS to neurodevelopmental disorders, including ASD and intellectual disability. In particular, several genes encoding DUBs have been associated with ASD, underscoring the importance of tightly regulated ubiquitination dynamics in brain development and synaptic plasticity [15,16]. In this context, *USP24*, a member of the ubiquitin-specific protease family, has recently emerged as a candidate gene of interest in a study conducted in a Qatari cohort [18], although its precise role in neurodevelopment and ASD pathogenesis remains to be elucidated.

In this study, we report a patient with a complex neurodevelopmental phenotype in whom trio-based WES identified a de novo variant in *USP24*, alongside inherited variants in *NRXN2* and *LRP2*. Given the emerging role of *USP24* in neurodevelopment and its functional relevance within the ubiquitin–proteasome system, this gene represents the most compelling candidate underlying the observed phenotype. This case provides additional insight into the complex genetic architecture of NDDs and supports a model involving a primary pathogenic event with potential modifying or additive effects.

## 2. Results

### 2.1. Clinical Report

The patient is the only child of non-consanguineous parents and was born at term via uncomplicated vaginal delivery following an uneventful pregnancy, with no reported perinatal complications (birth weight 3080 g; length 52 cm). A first cousin on the maternal side is affected by neurodevelopmental delay, and a paternal cousin presents with intellectual disability.

Early psychomotor development was overall within normal limits for independent walking (achieved at approximately 12 months), while sphincter control was acquired at around 3 years of age. First words emerged at approximately 12 months, followed by language regression at around 16 months, which led to initial hospitalization at the age of approximately 2 years and the initiation of rehabilitative interventions (speech therapy, psychomotor therapy, and cognitive behavioral therapy), with progressive improvement over time.

At approximately 6 years of age, the patient experienced her first seizure, characterized by absence episode, which recurred over a short period. She was subsequently hospitalized and started on antiepileptic treatment with valproic acid, which is ongoing and has resulted in good seizure control. No further significant seizures have been reported since 6 years of age. Brain MRI performed at approximately 8 years of age was unremarkable; repeated EEG recorded over time showed paroxysmal abnormalities localized over the frontal regions of the right hemisphere.

At 20 years of age, the patient underwent re-evaluation and was diagnosed with level 1 ASD, with fluent language and borderline intellectual functioning (BIF), in association with developmental coordination disorder and epilepsy. Array-CGH analysis performed during previous evaluations was normal. Currently, no significant behavioral disturbances or sleep disorders are reported; however, selective eating is noted.

On physical examination, general conditions are good. Macrocephaly is present (head circumference 58 cm, 97th percentile), while weight (52 kg, 25th percentile) and height (163 cm, 25th–50th percentile) are within normal ranges. No abnormalities were detected on general clinical examination.

Overall, the clinical picture is consistent with level 1 ASD, borderline intellectual functioning, developmental coordination disorder, and epilepsy.

### 2.2. WES Analysis

**Figure 1:**
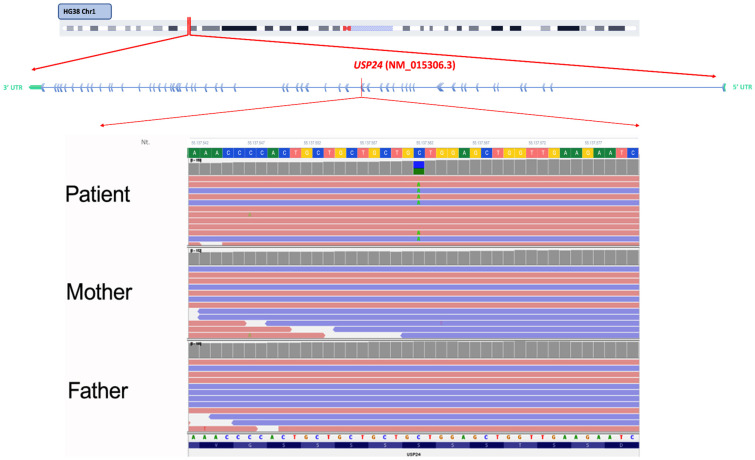
Integrative Genomics Viewer (IGV) visualization of the de novo variant c.3155G>T (p.Ser1052Ile) in the *USP24* (NM_015306.3) gene. The identified variant showed high confidence, with a variant quality score of 1662.64, a read depth of 146×, and a balanced allele distribution (C: 54.8%, A: 45.2%).

WES-Trio analysis prioritized three rare heterozygous variants located in the *USP24*, *NRXN2*, and *LRP2* genes. Among these, the variant c.3155G>T (p.Ser1052Ile) in the *USP24* (NM_015306.3) gene was identified as de novo, being absent in both parents (Figure 1).

Two additional variants of uncertain significance (VUS) were identified in *NRXN2* (NM_138732.3) and *LRP2* (NM_004525.3). The *NRXN2* variant c.388G>C (p.Gly130Arg) and the *LRP2* variant c.6395C>A (p.Ser2132Tyr) were both inherited from the clinically unaffected father (Figures S1 and S2). According to ACMG classification, the *NRXN2* variant meets criteria BP1 and PM2, whereas the *LRP2* variant meets BP1, PM2, and PP3.

No pathogenic or likely pathogenic copy number variants were detected by array-CGH analysis. Overall, WES highlighted the de novo *USP24* variant as the most relevant candidate contributing to the patient’s phenotype, while the inherited *NRXN2* and *LRP2* variants remain classified as VUS.

In silico prediction tools support a potential deleterious effect for all three variants. The *USP24* variant shows a SIFT score of 0.01, PolyPhen-2 score of 0.920, and CADD score of 26.6. All these three computational predictors supported the pathogenicity of the USP24 variant. The *NRXN2* variant presents a SIFT score of 0.004, supporting an uncertain significance. On the other hand, PolyPhen-2 score of 0.874, and CADD score of 28.1 supported the likely pathogenicity of the *NRXN2* variant. The *LRP2* variant is predicted to be damaging by PolyPhen-2 (0.999) and has a CADD score of 24.7, and was described as uncertain. In silico prediction scores for the identified variants are summarized in Table 1.

The *USP24* variant is absent from the gnomAD (Exome and Genome) database. Conservation analysis indicates that it lies within a highly conserved genomic region, with a GERP score of 5.16 and a PhyloP score of 7.388, supporting functional constraint at this site.

The *NRXN2* variant has a very low allele frequency (<0.01) in gnomAD and shows moderate evolutionary conservation (GERP score: 3.53), although the relatively low PhyloP value (1.152) suggests weaker conservation at the nucleotide level.

The *LRP2* variant is extremely rare (frequency <0.01% in gnomAD Exome and absent in gnomAD Genome) and is predicted to have a deleterious effect by multiple algorithms. It shows a high degree of conservation (GERP score: 6.03), indicating functional importance.

The DOMINO inheritance predictor classified *USP24* as having an intermediate probability of dominant inheritance (score = 0.478), whereas *NRXN2* and *LRP2* were predicted to follow an autosomal dominant inheritance pattern, with scores of 0.927 and 0.932, respectively.

### 2.3. Structural Prediction

**Figure 2:**
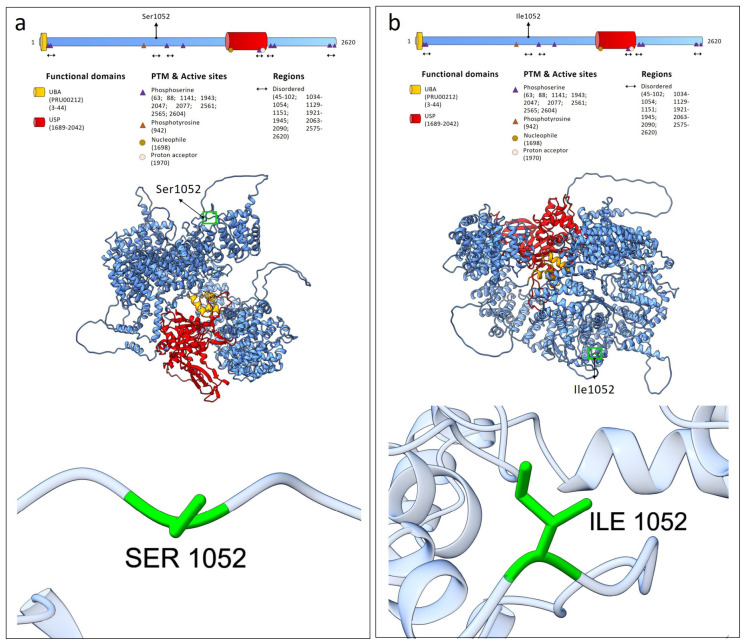
Structural analysis of the p.Ser1052Ile variant in USP24. (**a**) Domain organization of USP24, including UBA and USP domains, with the position of Ser1052 indicated. The predicted three-dimensional structure shows the localization of the residue outside major functional domains. A detailed view of Ser1052 is provided. (**b**) Structural model of the p.Ser1052Ile variant. The variant is located within the disordered region 1034–1054 annotated in the Uniprot database.

Structural modeling of the wild-type and p.Ser1052Ile mutant USP24 proteins showed that the wild-type structure contained 2268 hydrogen bonds in the best-ranked model, whereas the mutant protein exhibited 2266 hydrogen bonds. At the variant site, neither the wild-type residue (Ser1052) nor the mutant residue (Ile1052) was involved in hydrogen bond formation (Figure 2). Structural alignment between the wild-type and p.Ser1052Ile mutant USP24 proteins showed a high degree of similarity, with an RMSD of 0.953 Å calculated over 1915 pruned residues (4.531 Å across all 2620 residues).

To assess the impact of the p.Ser1052Ile mutation on protein stability, mCSM, DynaMut2, and MuPro were applied. All tools predicted a destabilizing effect associated with the variant. Table 2 summarizes the ΔΔG values (kcal·mol^−1^) for the USP24 mutation, along with the MuPro prediction score (ranging from −1 to 1).

Structural modeling of the wild-type and mutant proteins was performed using AlphaFold3 predictions. For NRXN2, the p.Gly130Arg variant did not significantly affect overall structure: both wild-type and mutant models showed a comparable number of hydrogen bonds (1119 vs. 1120), and no hydrogen bonds involved the residue at position 130 (Figure S3). The variant is located within the Laminin G-like 1 domain (residues 29–206). Structural alignment revealed high similarity, with 1050 aligned residues and an RMSD of 0.778 Å, while the higher RMSD observed for the full-length protein (37.756 Å) reflects divergence outside the aligned core. For LRP2, the p.Ser2132Tyr variant resulted in a local reduction in hydrogen bonding at the mutation site (four bonds in the wild-type vs. two in the mutant), while the overall number of hydrogen bonds remained comparable. The variant lies within the LDL-receptor class B 19 domain (residues 2107–2156) (Figure S4). Structural alignment showed high similarity in the aligned regions (815 residues; RMSD = 0.766 Å), whereas the higher RMSD for the full-length protein (44.687 Å) indicates structural divergence outside the aligned core.

Concerning the in silico analysis of protein flexibility, the IUPred2 profile revealed that the region surrounding residue 1052 lies within a partially disordered segment, with scores approaching or exceeding the 0.5 threshold (Figure S5).

Comparison between wild-type and p.Ser1052Ile USP24 proteins showed a consistent local decrease in disorder propensity in the mutant, particularly within the 1035–1060 region.

## 3. Discussion

In this study, we report a female patient with a broad spectrum of NDDs, including level 1 ASD, BIF, epilepsy. Family history reveals the presence of neurodevelopmental disorders in extended relatives. Although these cases occur in more distant relatives and their genetic causes are unknown, this observation may suggest a broader familial susceptibility to neurodevelopmental conditions. In the present case, chromosomal microarray analysis (array-CGH) did not reveal pathogenic copy number variations, supporting the hypothesis that single nucleotide variants may contribute to the patient’s phenotype. Trio-based WES identified three rare variants in the *NRXN2*, *LRP2*, and *USP24* genes that could potentially be involved in the clinical presentation.

The *NRXN2* (c.388G>C; p.Gly130Arg) and *LRP2* (c.6395C>A; p.Ser2132Tyr) variants were inherited from the unaffected father and are currently classified as variants of uncertain significance (VUS) according to ACMG guidelines. Although their paternal origin reduces the likelihood of a fully penetrant causal role, their contribution to the phenotype cannot be excluded. In particular, the *NRXN2* variant may act as a susceptibility factor within a multifactorial genetic background. *NRXN2* belongs to the neurexin gene family, whose members are characterized by incomplete penetrance, pleiotropy, and variable expressivity [19,20,21]. Variants in *NRXN1* are well-established risk factors for neurodevelopmental and neuropsychiatric disorders and frequently exhibit reduced penetrance. Given the functional similarity among neurexin genes and the growing evidence implicating *NRXN2* in neurodevelopmental disorders [22,23,24], the identified variant may contribute to the patient’s phenotype through an additive effect, despite being inherited from the clinically unaffected parent.

*LRP2* is known to be associated with Donnai–Barrow syndrome (MIM #222448), a rare autosomal recessive disorder characterized by craniofacial dysmorphisms, ocular anomalies, sensorineural hearing loss, agenesis of the corpus callosum, and global developmental delay. In the present case, the patient does not exhibit the typical craniofacial dysmorphisms associated with this syndrome. However, macrocephaly (97th percentile) was observed, which may represent a partially overlapping feature. Nevertheless, given the heterozygous state of the variant and the lack of other cardinal features of Donnai–Barrow syndrome, a direct causal role of the *LRP2* variant in the patient’s phenotype appears unlikely. Several studies have suggested a potential association between *LRP2* and ASD, hypothesizing that alterations in *LRP2* may impair Sonic Hedgehog (SHH) signaling pathways involved in neurodevelopment [25,26]. However, this association remains speculative and has not yet been conclusively demonstrated.

Conversely, the variant identified in the *USP24* gene (c.3155G>T; p.Ser1052Ile) arose de novo and was classified as likely pathogenic according to ACMG criteria. De novo variants are well recognized as important contributors to NDDs, including ASD and epilepsy [14,27,28]. Therefore, the *USP24* variant represents the most plausible candidate underlying the patient’s phenotype and may have a greater pathogenic impact compared with the inherited variants. The pathogenicity prediction tools CADD, PolyPhen-2, and SIFT supported the likely pathogenicity of the identified variant. Although the precise biological role of *USP24* in neurodevelopment remains to be fully elucidated, its involvement in ubiquitin-mediated cellular pathways suggests a potential role in neuronal development and synaptic regulation. *USP24* has been previously described as a risk and candidate gene for Parkinson’s disease [29,30,31] and, more recently, has been proposed as a potential risk gene for ASD in a genome sequencing study conducted in a Qatari cohort [18]. According to the ClinVar database, to date no pathogenic or likely pathogenic missense variants in USP24 have been reported. According to the RNAct database, several putative RNA targets of USP24 have been identified; among these, genes such as *CACNG8*, *HRAS*, *AUTS2*, *NRG1*, and MBP are known to be involved in neurodevelopmental processes [32,33,34,35,36]. Notably, some of these candidates (e.g., *CACNG8* and *HRAS*) display relatively high interaction scores and z-scores, suggesting a stronger predicted binding propensity. These genes are implicated in key pathways underlying synaptic transmission, neuronal differentiation, and brain maturation, thereby providing a potential mechanistic link between *USP24* function and neurodevelopmental phenotypes. However, it is important to emphasize that RNAct predictions are based on computational models of protein–RNA interactions and do not constitute direct experimental evidence. Therefore, while these findings suggest that USP24 may participate in RNA regulatory networks relevant to neurodevelopment, experimental studies are required to confirm the biological significance of these predicted interactions.

Although the de novo USP24 variant appears to represent the main genetic event underlying the phenotype, the potential contribution of the inherited *NRXN2* and *LRP2* variants cannot be excluded. Taken together, these findings support a complex genetic architecture, possibly consistent with a multifactorial or oligogenic model. All prioritized variants are missense changes. Notably, gene-level constraint metrics retrieved from DECIPHER database, further support their potential relevance. In fact, *USP24*, *LRP2*, and NRXN2 display missense Z-scores of 4.14, 4.54, and 3.1, respectively, indicating significant intolerance to missense variation. These findings suggest that amino acid substitutions in these genes are more likely to have functional consequences. It is worth mentioning that all these three genes are annotated as SFARI genes with a score of 1 for *NRXN2*, 2 for *LRP2* and 3 for USP24. Structural predictions analysis did not reveal notable structural changes in all the predicted models. Nevertheless, we would like to acknowledge that the analyzed models are structural predictions that did not reflect the functional consequences of the identified variants.

**Figure 3:**
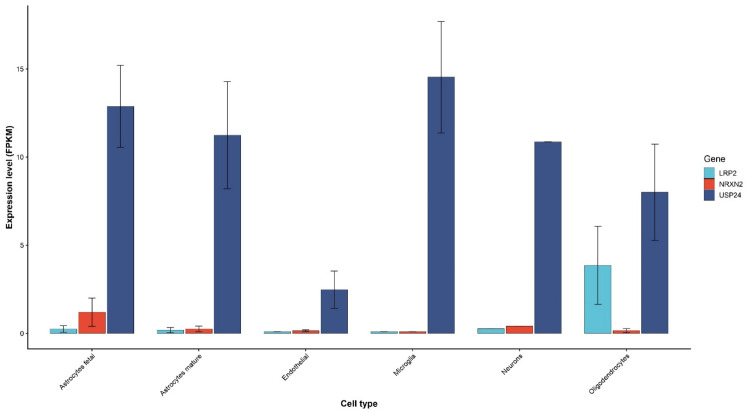
Expression profiles of *LRP2*, *NRXN2*, and *USP24* across human brain cell types. Bar plots represent the mean expression levels (FPKM) of *LRP2*, *NRXN2*, and *USP24* across different human brain cell populations, including fetal astrocytes, mature astrocytes, endothelial cells, microglia, neurons, and oligodendrocytes, as derived from the Brain RNA-seq database. Error bars indicate standard deviation (SD).

The analysis of the Gene Ontology (GO) annotations revealed a convergence of the identified genes on biological processes related to synaptic function and neuronal communication, including synapse assembly (GO:0007416), neurotransmitter secretion (GO:0007269), and trans-synaptic signaling (GO:0098820). Additional enrichment in pathways related to nervous system development (GO:0007399) and social behavior (GO:0035176) further supports their potential involvement in neurodevelopmental phenotypes. These findings suggest that, despite their distinct molecular functions, *USP24*, *NRXN2*, and *LRP2* may contribute to a shared biological network relevant to ASD and epilepsy. To further support this observation, it is noteworthy that all three genes are expressed in the human brain, although their expression is compartmentalized across different cell types and structures, as reported in the Brain RNA-seq database (Figure 3).

*USP24* shows relatively broad expression across all analyzed cell types, with higher levels in microglia, neurons, and astrocytes. *LRP2* displays prominent expression in oligodendrocytes, whereas *NRXN2* exhibits overall lower expression levels across all cell types. These data highlight distinct but partially overlapping expression patterns, supporting a potential contribution of these genes to diverse cellular components of the central nervous system.

**Figure 4:**
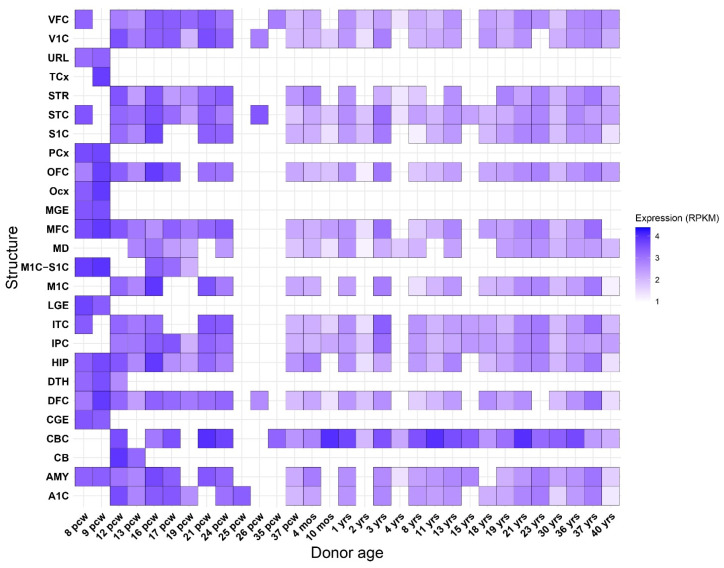
Developmental brain expression of *USP24*. Data was retrieved from BrainSpan database and are measured in RPKM. The abbreviation used were: primary auditory cortex (core) (A1C); amygdaloid complex (AMY); cerebellum (CB); cerebellar cortex (CBC); caudal ganglionic eminence (CGE); dorsolateral prefrontal cortex (DFC); dorsal thalamus (DTH); hippocampus (hippocampal formation) (HIP); posteroventral (inferior) parietal cortex (IPC); inferolateral temporal cortex (area TEv, area 20) (ITC); lateral ganglionic eminence (LGE); primary motor cortex (area M1, area 4) (M1C); primary motor-sensory cortex (samples) (M1C-S1C); mediodorsal nucleus of thalamus (MD); anterior (rostral) cingulate (medial prefrontal) cortex (MFC); medial ganglionic eminence (MGE); occipital neocortex (Ocx); orbital frontal cortex (OFC); parietal neocortex (PCx); primary somatosensory cortex (area S1, areas 3,1,2) (S1C); posterior (caudal) superior temporal cortex (area 22c) (STC); striatum (STR); temporal neocortex (TCx); upper (rostral) rhombic lip (URL); primary visual cortex (striate cortex, area V1/17) (V1C); ventrolateral prefrontal cortex (VFC); post-conceptional week (pcw); months (mos); years (yrs).

For providing further insights related to the *USP24* expression form human brain, we retrieved the brain developmental expression data from BrainSpan database (Figure 4).

The expression profile of *USP24* across brain development highlights a clear temporal and spatial pattern. *USP24* shows robust expression during early developmental stages, particularly across multiple brain regions, suggesting a potential role in neurodevelopmental processes.

Notably, high expression is observed during prenatal and early postnatal periods, which are characterized by intense cellular events such as neuronal differentiation, migration, and synaptogenesis. This temporal enrichment supports the hypothesis that *USP24* may contribute to the regulation of these processes, possibly through its function as a deubiquitinating enzyme involved in protein turnover and signaling pathways. This persistent expression suggests that, beyond development, USP24 may also play a role in maintaining neuronal function or circuit stability in the mature brain. Although USP24 is not directly involved in synaptic transmission, it encodes a deubiquitinating enzyme that regulates protein turnover through the ubiquitin–proteasome system, a pathway essential for synaptic function and plasticity. Dysregulation of this system may affect the stability and trafficking of synaptic proteins [7,37,38], thereby indirectly influencing neuronal communication and providing a mechanistic link between USP24 and the synaptic processes highlighted by the functional annotation analysis.

Concerning the in silico structural predictions, only subtle differences were observed between the wild-type and the p.Ser1052Ile variant of USP24. Specifically, AlphaFold3 predicted minor local variations in the three-dimensional protein model. Additionally, the computational protein stability predictors mCSM, DynaMut2, and MuPro indicated a destabilizing effect of the mutation on the protein structure compared to the wild-type. We acknowledge that in silico predictions should be interpreted with caution and considered as supportive rather than definitive evidence.

The p.Ser1052Ile mutation of USP24 identified in this study is located within the disordered region (1034–1054 aa) annotated in the Uniprot database. Consistently, IUPred2 analysis indicated a reduction in disorder propensity at the mutation site. From a biochemical perspective, the substitution of serine with isoleucine at position 1052 introduces a significant change in physicochemical properties, replacing a small polar residue with a bulkier hydrophobic one. This change is consistent with the observed decrease in disorder, as hydrophobic residues generally promote local structural stabilization. Importantly, the affected region lies within a predicted intrinsically disordered segment, which is likely involved in transient protein–protein interactions. Intrinsically disordered regions (IDRs) are known to play key roles in regulatory processes, often through short linear motifs or via folding upon binding [39,40]. Despite lacking a well-defined three-dimensional structure, they are pivotal for cellular processes ranging from transcriptional control and cell signaling to subcellular organization [39,40].

Comparative sequence analysis demonstrated that USP24 is highly conserved across multiple mammalian species, as evidenced by the high pairwise identity observed in the heatmap and clustering analysis (Figure S6). Notably, the residue affected by the variant, Ser1052, is strictly conserved across all examined orthologues. This high degree of evolutionary conservation suggests that this position is functionally important and less tolerant to variation, supporting the potential pathogenic relevance of the identified variant.

Based on these findings, our results are consistent with a potential cumulative contribution of the identified variants in *NRXN2*, *LRP2*, and *USP24* to the patient’s phenotype. Among them, the de novo *USP24* variant is likely to represent the primary genetic event, given its mutational origin and predicted pathogenicity. However, the contribution of the inherited variants cannot be excluded. In particular, the *NRXN2* variant may be consistent with mechanisms such as incomplete penetrance or variable expressivity, while the *LRP2* variant could act as a modifying factor. Furthermore, the presence of additional genetic alterations not detectable by array-CGH or WES cannot be ruled out. We emphasize that the de novo occurrence of the USP24 variant supports its prioritization as the main candidate gene. ORVAL analysis identified a high-confidence digenic interaction between NRXN2 and LRP2 (VarCoPP score = 0.9175), while combinations involving USP24 showed slightly lower but still significant scores (USP24–LRP2: 0.8425; NRXN2–USP24: 0.7225), consistent with a potential modifier role. Together, these findings suggest that the phenotype may be shaped by an oligogenic background involving inherited variants in NRXN2 and LRP2. Overall, this study does not demonstrate an oligogenic mechanism but rather identifies *USP24* as a primary candidate gene, supported by its de novo occurrence and consistency with the clinical phenotype. While additional rare variants in *LRP2* and *NRXN2* were identified, their contribution remains uncertain, particularly given their inheritance from unaffected parents. Therefore, our findings should be considered hypothesis-generating, suggesting a possible but unproven cumulative contribution of multiple variants in neurodevelopmental disorders. Importantly, the absence of functional validation limits causal inference. Further experimental studies will be required to clarify the role of USP24 and to determine whether additional variants may have a modifying effect on the phenotype.

## 4. Materials and Methods

### 4.1. Whole-Exome Sequencing

Genomic DNA was obtained from peripheral blood leukocytes of the patient and both parents, according to a previously described method [41]. Library preparation for the Trio-based WES analysis was carried out using the Agilent SureSelect V7 Kit (Santa Clara, CA, USA), according to the manufacturer’s protocol. The sequencing run was performed using the Illumina HiSeq 3000 (San Diego, CA, USA). Sequencing reads were aligned to the human reference genome (GRCh38) using BWA-MEM, and variant calling was performed with GATK HaplotypeCaller following best-practice recommendations. Variant annotation was carried out using ANNOVAR (release 29 July 2025). Quality control and filtering were applied in a stepwise manner. Variants with low sequencing support were excluded, retaining those with a minimum read depth ≥ 20× (covering ≥97% of targeted regions) and genotype quality (GQ) ≥ 20. Allele balance was required to be between 0.05 and 1.0 to retain both heterozygous and homozygous variants while excluding likely sequencing artifacts. Variants were then filtered based on predicted functional consequence, retaining exonic and canonical splice-site variants and excluding synonymous variants without predicted splice impact and non-coding variants. Population frequency filtering was applied independently using public databases (gnomAD v4.1.0, 1000 Genomes Project, ExAC, and ESP6500; accessed on 9 March 2026), removing variants with a minor allele frequency (MAF) ≥ 1%. This threshold was used as an initial screening criterion to account for population-specific genetic variability, followed by prioritization of variants that were absent or extremely rare in population databases. Finally, candidate variants were prioritized according to presumed inheritance patterns (recessive, de novo, or X-linked), predicted functional impact, and consistency with the clinical phenotype described using Human Phenotype Ontology (HPO) terms, including hypergammaglobulinemia (HP:0002960), hypoglycemia (HP:0001943), hyperferremia (HP:0003281), elevated alanine level (HP:0003348), hypoalbuminemia (HP:0003073), exophoria (HP:0000643), renal cyst (HP:0000107), macrocephaly (HP:0000256), feeding difficulties (HP:0011968), borderline intellectual disability (HP:0006889), ataxia (HP:0002315), autism (HP:0000717), and seizures (HP:0001250). All prioritized variants were subsequently validated by conventional Sanger sequencing. The reaction was performed with the BigDye™ Terminator v1.1 Cycle Sequencing Kit (Life Technologies, Carlsbad, CA, USA) on the SeqStudio Genetic Analyzer (Thermo Fisher Scientific, Waltham, MA, USA).

### 4.2. Data Analysis

Pathogenic variants were searched on The Human Gene Mutation Database (HGMD Professional 2025.2) (accessed on 9 March 2026). Franklin by QIAGEN (Hilden, Germany) was used for filtering and prioritizing the genetic variants from the VCF file. The identified variant was classified using the “American College of Medical Genetics” (ACMG) guidelines and criteria [42]. The ClinVar database (https://www.ncbi.nlm.nih.gov/clinvar) (accessed on 9 March 2026) was queried to identify genetic variants in *USP24*, *NRXN2* and *LRP2* genes. DOMINO [43] (https://domino.iob.ch/) (accessed on 9 March 2026) was used for predicting the inheritance pattern of the *USP24*, *NRXN2* and *LRP2* genes, adopting a scoring system ranging from 0 (autosomal recessive) to 1 (autosomal dominant). Pathogenecity predictors CADD [44], PolyPhen-2 (http://genetics.bwh.harvard.edu/pph2/) (accessed on 9 March 2026) and SIFT [45] have been used for predicting the pathogenecity significance of the USP24, NRXN2 and LRP2 variants. The RNAct database (https://rnact.tartaglialab.com/) (accessed on 9 March 2026) was used to identify RNAs interacting with USP24 based on computational predictions. The UniProt database (https://www.uniprot.org/) (accessed on 9 March 2026) was used to analyze protein domain organization. The AlphaFold3 server (https://alphafoldserver.com/) (accessed on 9 March 2026) was used to predict the structural effects of the identified variants in USP24, NRXN2, and LRP2. For each protein, the best-ranked model was selected based on the highest predicted local distance difference test (pLDDT) score. Structural prediction was performed for both the wild-type and mutated proteins. Protein structural analyses and visualization were performed using UCSF ChimeraX version 1.10.1. Protein stability changes associated with the p.Ser1052Ile variant in the USP24 gene were predicted using the computational tools mCSM (https://biosig.lab.uq.edu.au/mcsm/stability) (accessed on 9 March 2026), DynaMut2 (https://biosig.lab.uq.edu.au/dynamut2/) (accessed on 9 March 2026), and MuPro (https://mupro.proteomics.ics.uci.edu/) (accessed on 9 March 2026). Both mCSM and DynaMut2 estimate stability changes as ΔΔG values (kcal·mol^−1^), where negative values indicate a destabilizing effect. In contrast, MuPro provides a score ranging from −1 to 1, with negative values indicating decreased stability and positive values indicating increased stability. IUPred2 (https://iupred2a.elte.hu/) (accessed on 9 March 2026) was used to predict the effect of the p.Ser1052Ile variant on protein flexibility and intrinsic disorder in USP24. IUPred2 assigns a score to each residue ranging from 0 to 1, where lower scores indicate ordered regions and higher scores indicate intrinsically disordered regions. Clustal W USP24 protein alignment was carried out using the Bioedit software version 7.7. Oligogenic Resource for Variant Analysis (ORVAL) (https://orval.ibsquare.be/; accessed on 24 April 2026) was used to estimate the potential effects of disease-causing oligogenic variant combinations.

## Figures and Tables

**Table 1:** In silico pathogenicity predictions for the identified variants in *USP24*, *NRXN2*, and *LRP2*. SIFT, PolyPhen-2, and CADD scores are reported. SIFT scores ≤0.05 indicate deleterious variants; PolyPhen-2 scores closer to 1.0 suggest a higher probability of a damaging effect; higher CADD scores indicate greater predicted deleteriousness.

	*USP24*	*NRXN2*	*LRP2*
SIFT	0.01	0.004	-
PolyPhen2	0.920	0.874	0.999
CADD	26.6	28.1	24.7

**Table 2:** Predicted effects of the p.Ser1052Ile variant in USP24 on protein stability. ΔΔG values (kcal mol^−1^) were calculated using mCSM and DynaMut2, while MuPro provides a confidence score indicating the direction of stability change. Negative ΔΔG values and MuPro scores are consistent with a destabilizing effect.

	ΔΔG
mCSM	−0.564 kcal mol^−1^
DynaMut2	−0.64 kcal mol^−1^
MuPro	−0.311 score

## Data Availability

The data presented in this study are available upon request from the corresponding author.

## Supplementary Materials

The following supporting information can be downloaded at: https://www.mdpi.com/article/10.3390/ijms27094086/s1.

## Abbreviations

The following abbreviations are used in this manuscript:

ACMG	American College of Medical Genetics and Genomics
ASD	Autism spectrum disorder
BIF	Borderline intellectual functioning
DSM-5	Diagnostic and Statistical Manual of Mental Disorders, Fifth Edition
DUB	Deubiquitinating enzyme
GO	Gene ontology
IDR	Intrinsically disordered region
NDD	Neurodevelopmental disorder
pLDDT	predicted local distance difference test
RMSD	Root mean square deviation
RPKM	Reads Per Kilobase Million
SD	Standard deviation
VUS	Variant of uncertain significance
WES	Whole-exome sequencing
