# Supplementary material for: A De Novo USP24 Variant as a Candidate Driver in a Neurodevelopmental Disorder: Insights from Trio-Based Whole-Exome Sequencing
Source: Int J Mol Sci. 2026 May 2;27(9):4086. doi: 10.3390/ijms27094086 (PMC13164258; doi:10.3390/ijms27094086)
Supplement: Supplementary file 1 [file ijms-27-04086-s001.zip › ijms-4275362-supplementary.pdf]

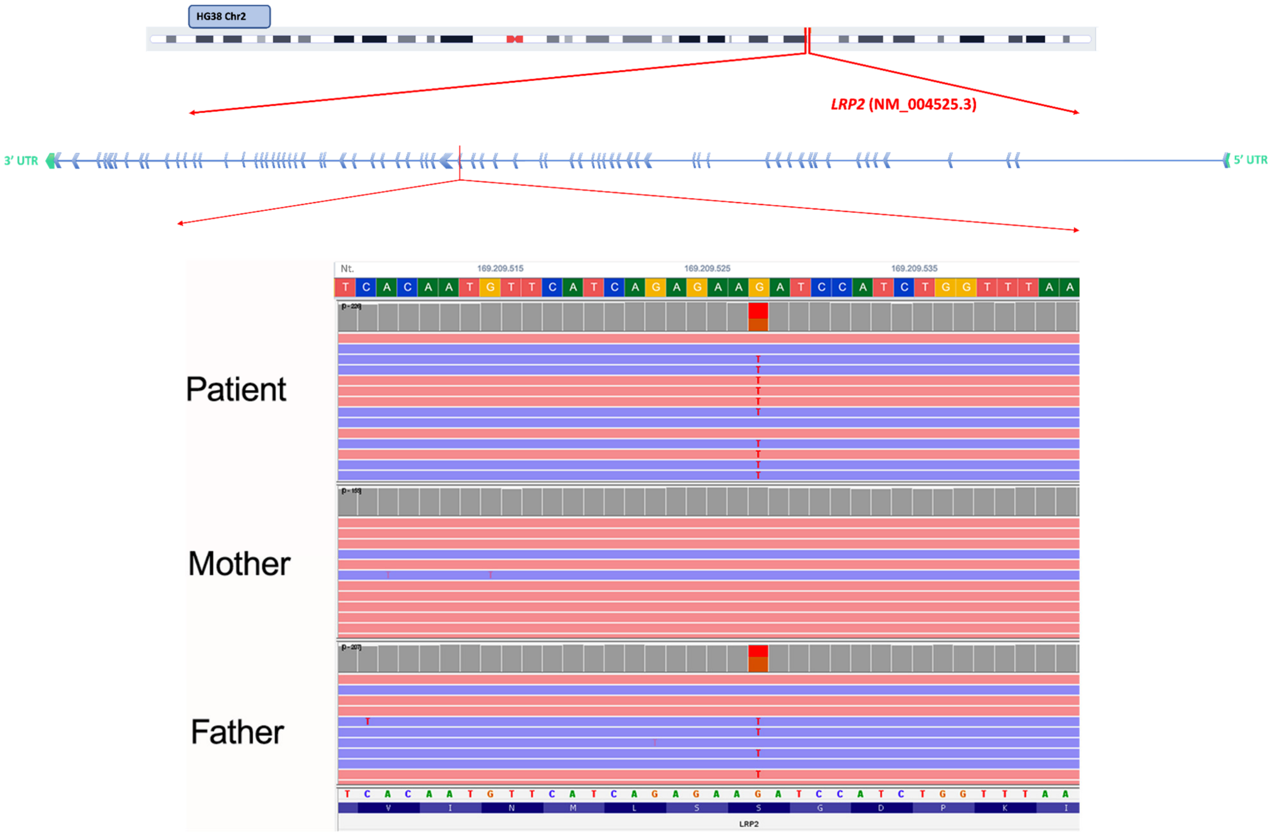

Figure S1. IGV visualization of the variant c.6395C>A (p.Ser2132Tyr) in the LRP2 (NM_004525.3) gene, inherited from the patient's father.

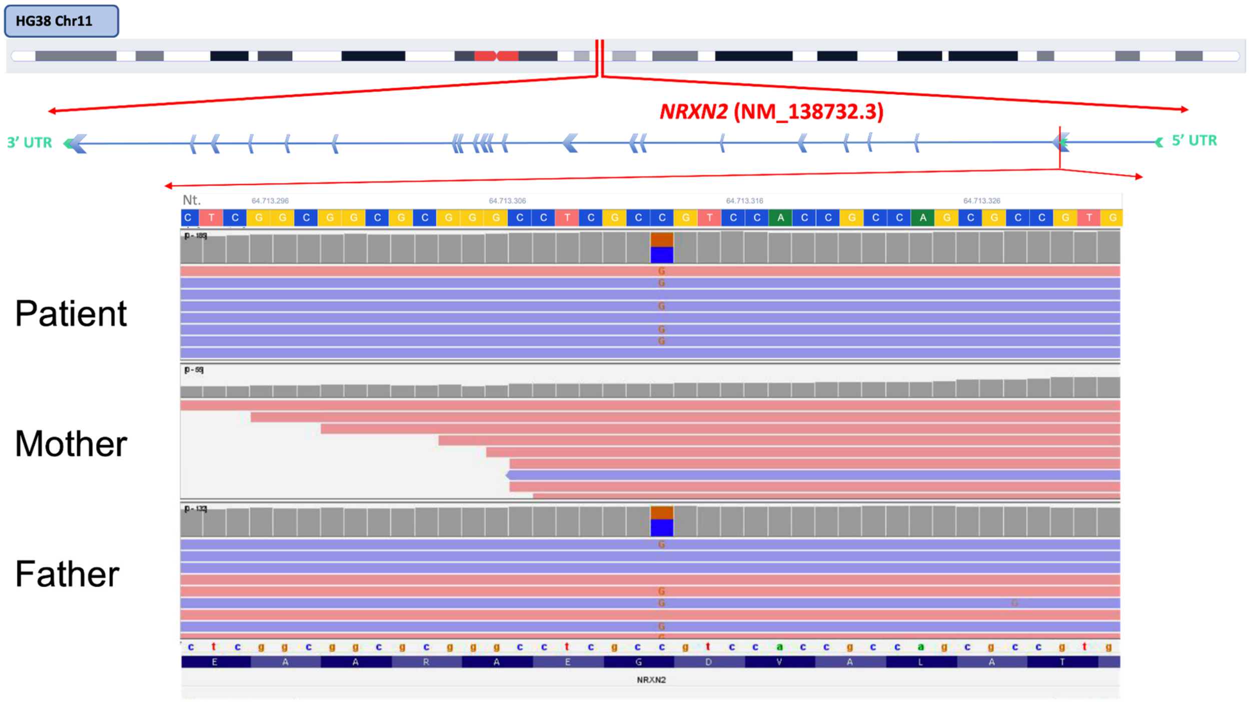

Figure S2. IGV visualization of the variant c.388G>C (p.Gly130Arg) in *NRXN2* (NM_138732.3) gene.

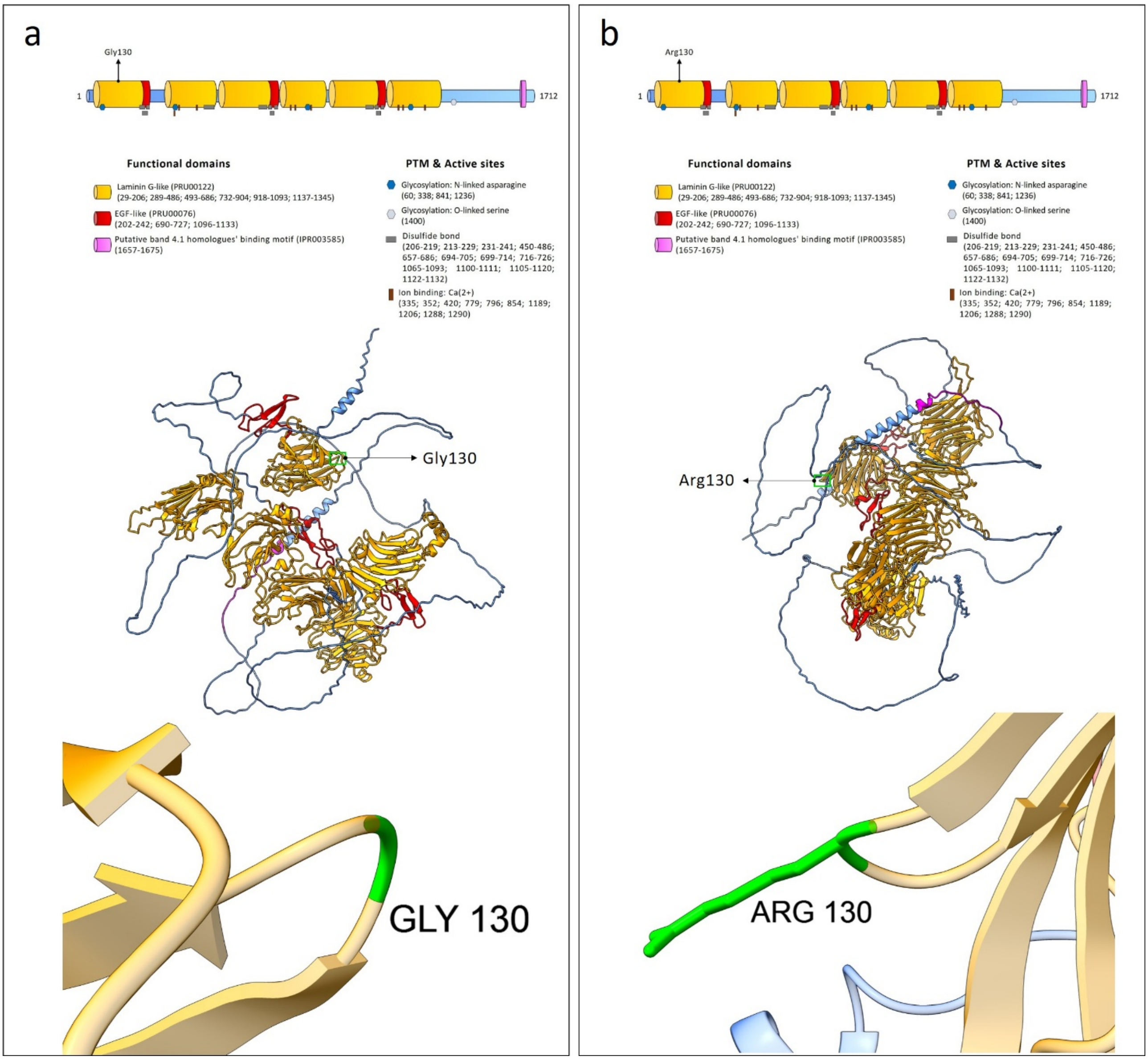

Figure S3. Structural analysis of the p.Gly130Arg variant in NRXN2. (a) Schematic representation of NRXN2 protein domains, including Laminin G-like and EGF-like domains, with the position of Gly130 indicated. The predicted structure shows the residue localization, and a close-up view highlights Gly130 within the protein context. (b) Structural model of the p.Gly130Arg variant. The substitution introduces a bulkier residue.

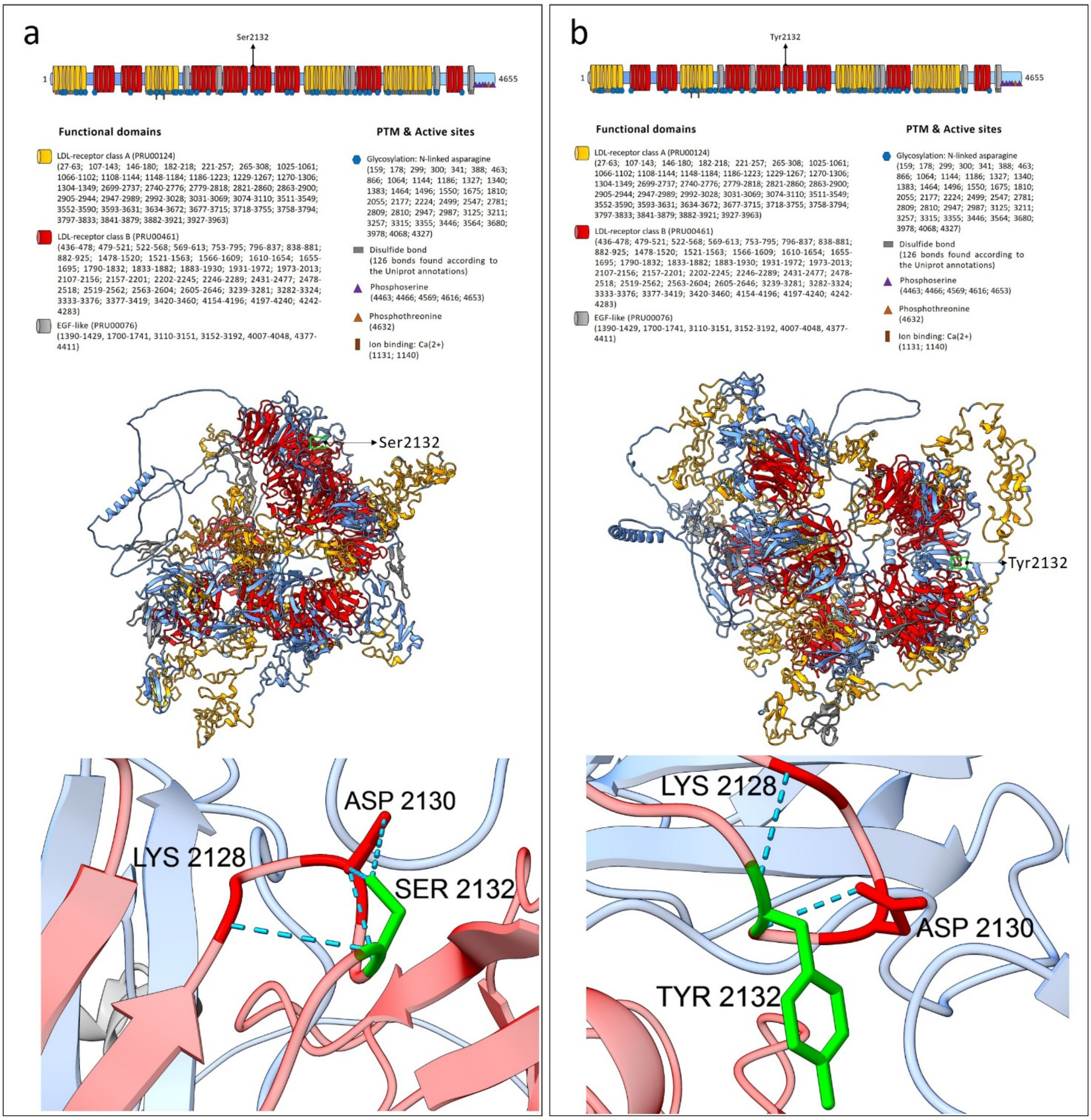

Figure S4. Structural analysis of the p.Ser2132Tyr variant in LRP2. (a) Schematic representation of the LRP2 protein showing functional domains and post-translational modification (PTM) sites, with the position of Ser2132 indicated. The predicted three-dimensional structure highlights the localization of the residue within the protein. A close-up view shows Ser2132 and its interactions with neighboring residues. (b) Corresponding analysis of the mutant protein (p.Ser2132Tyr). The substitution results in a reduction of hydrogen bond interactions at the variant site, as illustrated in the zoomed-in view.

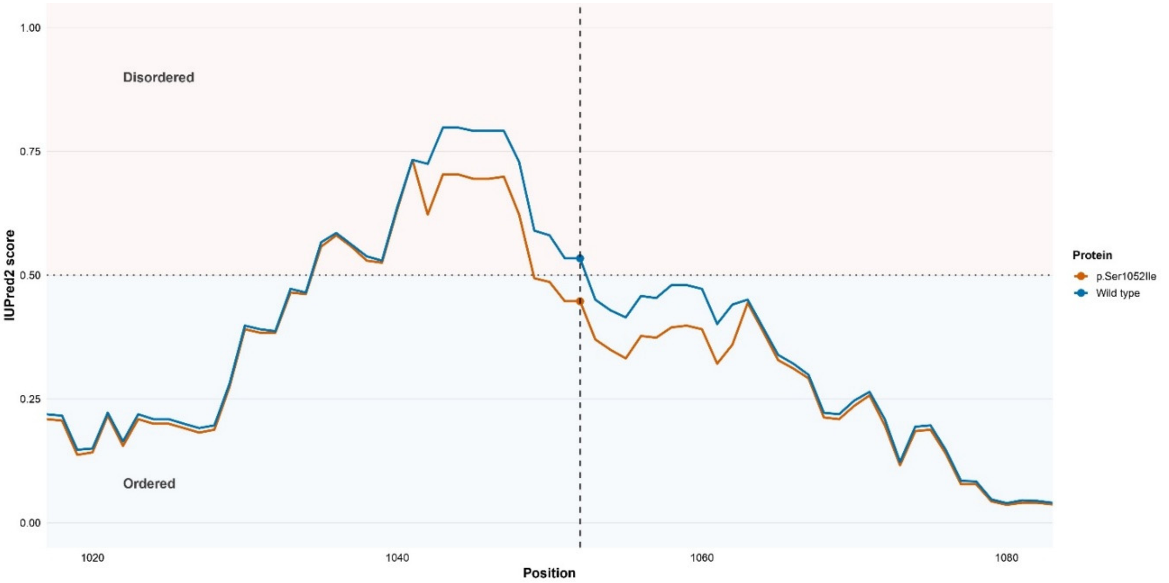

Figure S5. IUPred2 prediction of intrinsic disorder for the USP24 wild-type and p.Ser1052Ile variant. The per-residue disorder score (0–1) is plotted across the protein region, with the threshold for disorder indicated at 0.5. The dashed vertical line marks the position of the variant (Ser1052). Both wild-type and mutant profiles show similar trends, although a slight reduction in disorder propensity is observed in the mutant around the variant site.

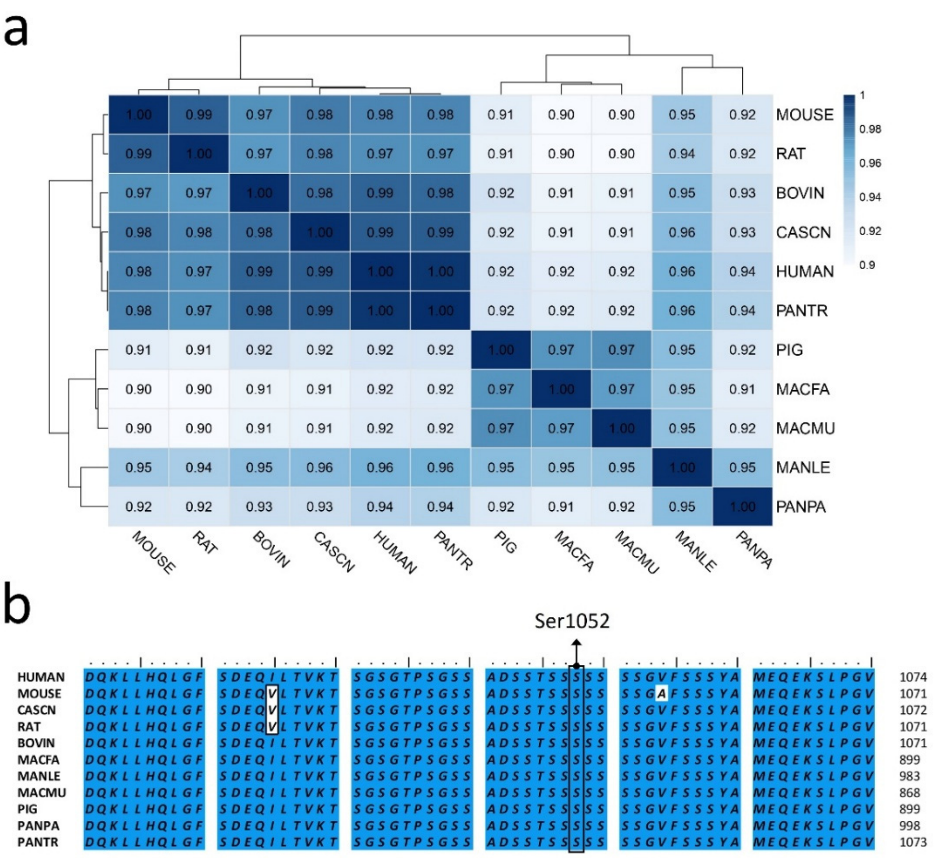

Figure S6. Evolutionary conservation of USP24 across mammalian species. (a) Pairwise sequence identity heatmap and hierarchical clustering of USP24 orthologs across different mammals, showing a high degree of sequence conservation. (b) Multiple sequence alignment of the USP24 protein region surrounding residue

Ser1052. The variant site is indicated by an arrow and boxed region. The Ser1052 residue is highly conserved across all analyzed species, supporting its potential functional relevance. Species abbreviations: MOUSE (*Mus musculus*), RAT (*Rattus norvegicus*), BOVIN (*Bos taurus*), CASCN (*Castor canadensis*), HUMAN (*Homo sapiens*), PANTR (*Pan troglodytes*), PIG (*Sus scrofa*), MACFA (*Macaca fascicularis*), MACMU (*Macaca mulatta*), MANLE (*Mandrillus leucophaeus*), PANPA (*Pan paniscus*).
